# Some Observations on Gastric and Duodenal Ulcer*Read at meeting of American Gastro-enterologic Assn., Atlantic City, June 1, 1917.

**Published:** 1917-12

**Authors:** J. C. Johnson

**Affiliations:** Atlanta, Ga.


### BUFFALO MEDICAL JOURNAL

Yearly Volume 73 DECEMBER, 1917 Number 5

### ORIGINAL ARTICLES

The right is reserved to decline papers not dealing with practical medical and surgical subjects, and such as might offend or fail to interest readers. Contributors are solely responsible for opinions, methods of expression and revision of proof.

### Some Observations on Gastric and Duodenal Ulcer.*

By J. C. JOHNSON, M. D., Atlanta, Ga.

Several years ago we published a review of forty cases of Ulcer. In this review we referred especially to the frequent association of ulcer with hyperhlorhydria, hypersecretion and gastrosucchorea, and endeavored to draw some conclusions as to the relation between them all. It appeared that there was no symptom common to these disorders always present, and no condition in any which might be considered pathognomonic of ulcer. But we believed then, and still are of the opinion, that ulcer is no more an entity than is any other disease of the stomach or duodenum, and that even as a clinical entity its existence may be questioned. However, before an intelligent idea can be formed as to the relation between two or more concurrent diesases we must first have definite knowledge of each separate disease, and must also be sure that what is classed as disease is something more than a phase or an epoch of disease.

In any event a study of Ulcer cannot be comprehensive which does not take into account each distinct process which may predispose to it or which may be present with it.

Experimental production of Ulcer has taught us much of value. But experiment cannot prove everything about anything. Art cannot create the conditions which develop as a sequence of orderly physiological perversion.

Surgery has given us a closer view of the morbid anatomy of Ulcer and of the deformities which often follow it. But

*Read at meeting of American Gastro-enterologic Assn., Atlantic City, June 1, 1917.

surgery can never determine its cause nor depict the tissue change incident to its development. Fortunately, there are other ways of knowing things than by seeing them, and other ways of finding out than by probing. The very problems which call for radical action often escape the eye and are discernible only to reason and common sense.

The truth about ulcer has its foundation in the Stomach and Duodenum themselves, and it can only be expressed in terms of pathology most intimately related to their functions. Upon this presumption we have tabulated other cases, fifty in number*' in order to enlarge our data and add something to our knowledge of this very common and very serious affection.

It may be noticed that six or twelve per cent. of these patients were under twenty years of age.

Seventeen, or thirty-four per cent., between twenty and thirty.

Fourteen, or twenty-eight per cent., between thirty and forty.

Ten, or twenty per cent., between forty and fifty.

Three, or six per cent., between fifty and sixty.

Three, or six per cent., between sixty and seventy.

Thirty, or sixty per cent., were males. Twenty, or forty per cent., were females.

Various occupations were represented.

The history of previous indigestion varied from six months to a life time.

The highest Hgb. was 88. The lowest 60. Highest B. P. 195; lowest 90.

In fourteen, or twenty-eight per cent., the greater curvature of the stomach was below the umbilicus—in some instances reaching almost to the pubis.

In eighteen, or thirty-six per cent., the greater curvature extended to the umbilicus.

*Of these, 28 were Duodenal, 16 Gastric, and 6 Pyloric.

In eighteen, or thirty-six per cent., the position and size of the stomach were apparently normal.

Thirty-nine, or seventy-eight per cent., were constipated.

In nine, or eighteen per cent., there was a daily stool.

Two, or four per cent., had diarrhea.

Local tenderness was present in thirty-nine, or seventy-eight per cent. In thirty, or sixty per cent., the tenderness or soreness was referred to the Epigastric region. In four, or eight per cent., to the right hypochondrium. In two, or four per cent., to the umbilicus.

*Of these, 28 were Duodenal, 16 Gastric, and 6 Pyloric.

In two, or four per cent., to the left hypochondrium. In one, or two per cent., to the Xiphoid angle.

Next in frequency to soreness was pain,—present in thirty, or sixty-eight per cent. In twenty-three, or forty-six per to the right hypochondrium, epigastrium and left hypo-per cent., to the pectoral region. In two, or four per cent., to the right hypochondrium. Epigastrium and left hypochondrium. In two, or four per cent., to the left iliac. In one each, or two per cent., respectively to the left hypochondrium, Xiphoid angle, umbilical regions.

Nausea was present in fifteen, or thirty per cent. -Vomiting in twelve, or twenty-four per cent. Hemorrhage was rare. Occult fecal was positive in six, or twelve per cent.

In twenty-one, or forty-two per cent., there was evidence of family tendency to gastro-intestinal disorder.

Four were members of the same family—including father, mother, and two sons.

In twenty-four, or forty-eight per cent., the history of family tendency was negative. In the remaining five, or ten per cent., the history was indefinite.

Six, or twelve per cent., had appendectomy, prior to examination, one or more as long as seven years.

In one the gall bladder had been drained. In one tonsils had been removed.

The extent of the examination and the methods used in each case are suggested by the table. Besides, no effort was spared to discover everything in the history and general condition of the patient that could have any bearing on the ulcer. Only that which we have considered pertinent is included in this report.

We do not agree with those who say that a diagnosis of ulcer can always be made by telephone or by mail. Ulcer is sometimes present when it is not suspected, and sometimes suspected when it is absent.

We are not sure that the most careful surgeon may not overlook an ulcer when he is looking for it. All ulcers are not the same in size, site, or period of development. Positive diagnosis of ulcer can be based only upon finding one.

There are some who say that the stomach tube, the laboratory and other means of diagnosis employed by the internist are unreliable and of little value. The ground upon which these observers stand is so remote from this particular field of scientific progress, they hear no sound from without but the echo of their own voices. They see nothing beyond the horizon of their own sphere. They are so firmly fixed in their opinions by the centripetal nature of their thoughts no ideas but their own can have accession, persuasion or cohesive power.

We would pass this point unnoticed did it not quicken us to reaffirm our faith in those verities which they deny without first having knowledge of them. The science of medicine is too deep and too broad for any man however tall to stand upon its bottom anywhere and reach from one extreme to the other.

Unless one has had a wide experience in certain lines of work by what authority may he speak so assuredly of what can and cannot be accomplished by other methods than his own. By what inspiration can he know what it has required years of arduous labor for others to attain.

It is inconsistent to say the least of it to speak of the danger of cancer from ulcer as a base and of the need of early diagnosis, and at the same time to decry the means we have without supplying something better, or even suggesting something just as good.

It is not easy to distinguish between gastric and duodenal ulcer in every case. Pain is not invariably present in either, nor does it always occur at a time so definite that a diagnosis may be based upon this symptom alone. The point of local tenderness in both also varies, as does the pain, according to the position of the stomach and duodenum, and the character and extent of functional disturbance. Even with malignant ulcer far advanced there may be an absence of both pain and soreness.

Stenosis alone is not diagnostic of ulcer, neither does it always enable us to locate one when it exists. Only by determination of all the conditions present can the necessary knowledge and significance of ulcer be obtained, and the needs of the patient be intelligently supplied. There is no symptom so slight, or seemingly without value which may with reason be disregarded.

For location of ulcer there is nothing more reliable than the Einhorn Thread Test, and for detection when in doubt it is of equal service.

The X-ray will not picture an ulcer in its early stages or before a crater has developed. On the other hand it may show a deformity resembling that due to ulcer, and yet the trouble may be one in which the stomach is not primarily involved.

The table shows that in 94% the Thread was positive, whereas the X-ray when used was positive in 38%.

The Thread may not be positive for ulcer on the greater curvature towards the splenic end or where the ulcer has healed, or is healing.

There is no theory of ulcer convertible into fact which detaches its formation from the histological and biological forces of normal tissue change.

We have before made occasion to express the belief that the primary causes of disease are overwork, underwork, injury and infection. If this classification is correct, it narrows the field of our present investigation and simplifies the result.

Fatigue of any organ predisposes to disease, even if it may not constitute a condition which within itself is technically disease. Certainly, it is often a controlling factor in the conduct of disease. The predisposition created by fatigue rests essentially in impairment of nutrition. But it is misleading and confusing to regard nutrition as a process. It is not a process, but a condition the result of many processes—and hence cannot be the cause primarily of any other process.

Fatigue of the stomach may be the result of effort to overcome resistance in some other organ to which it is intimately related, as the duodenum, liver, or heart. It may follow excessive functioning stimulated or required by errors in diet. It may also develop from continued exercise within normal limits, but with insufficient material for repair. The duodenum may suffer in a similar way from similar causes.

It is significant that in 42% of the patients there was a family history of gastro-intestinal disorder, and that four of the patients were father, mother and two sons. This suggests that a certain habit of metabolism, or state of nutrition predisposes to ulcer. Yet in others this history is lacking or indefinite. Whether ulcer be local or constitutional in origin, somatic metabolism as a causative factor however potent and essential must be more or less indirect. And even if essential, and the primary importance of gastric and duodenal function is denied, it remains to be explained why these organs and not others are selected for the local manifestation of the general disease.

Traumatism predisposes to actual disease, but unless the continuity of the tissue is disturbed to the extent that healing is impossible, disease does not follow. Even when the function of the tissue has been impaired before the injury is received, it resumes its former tone and efficiency when the disorganization from the injury has been overcome—unless infection supervenes. When infection is added to traumatism, or occurs independently, the morphological changes which follow do not represent all the characteristics of necrosis and reformation found in classical ulcer. Furthermore, before infection can occur, can produce, or become active in a morbid process there must pre-exist histological and functional condition of the tissue not normally a part or product of organic life,—unless there is possessed by some an immunity which is lacking in others. So far, we have nothing upon which to build a theory of immunity from ulcer.

The presence of Hcl. in the stomach is not favorable to pathogenic bacteria. Even when Hcl. is absent the protective power of normal gastric protoplasm may remain and repel invasion by micro organisms. This is often seen in Achylia Gastrica. If it were a fact that the protoplasm cannot be normal with Hcl. absent, it would only lend support to our contention that the majority of gastro-intestinal diseases are primary in their essential nature, and that ulcer as a feature is not the result of any single cause but of an association of causes. But in a majority of ulcer patients there is an excess of Hcl.—which leaves no room for argument that general impairment of gastric protoplasm is either a singular cause or a result of ulcer. Neither does it add anything to the theory of infection. Especially untenable is the idea advanced by some that ulcer is not only due primarily to infection, but that the appendix is the source of the infection. We found ulcer in 7 or 14% of the cases months or years after the appendix had been removed, and after other so called surgical lesions had been repaired. It is evident that the ulcer in these cases was independent of the appendix or was overlooked at the time of the operation. In either event, the need of more comprehensive knowledge and accurate diagnosis of gastro-intestinal diseases is hereby clearly demonstrated.

Much of the fallacy in modern teaching has its source in efforts to build the whole truth out of half the substance. Ulcer is not a ready made, hard and dried disease, but a gradual outgrowth of successive perversions in nutritive and functional activity, and only by appreciation of this fact and by proper employment of the means which it supplies can the best results from treatment be obtained.

Internists know that ulcers do get well without the knife, and that after many years these patients have all the appearance and comfort of perfect health. On the other hand, it is true that there is no successful treatment for some ulcers, or for the complications which they bring about, except by operation. But the ideal operation for ulcer will be selected only when the surgeon keeps before him the complex relation of the alimentary functions, and the more complex forces which make possible and maintain these functions normally, and brings all this to bear in interpreting the particular requirements in the field of operation. A true surgeon always does this. His task is made easier and his burden lighter if aided by the previous observations and present counsel of the internist. Their combined intelligence is not too much for any case.

For lack of these considerations or for lack of opportunity to apply them, operations often fail not only to restore to normal the parts primarily affected, but also fail to preserve that measure of compensatory and reciprocal action which has remained despite the disease.

---

**Typhoid Treated With Gay-Claypole Vaccine.** Frederick C. Gay of Berkeley, Calif., Jour. of Lab. and Clin. Med., Aug., reports a series of 98 cases with 7 deaths. The vaccine is used intravenously and is a multivalent, sensitized sediment. One-third of the cases showed a critical defervescence after the first or second injection and a permanent normal within a week. In most of the others, marked improvement was shown and no untoward results ascribable to the method were observed. (Note: 75 years ago, when antivariolar vaccination was the only one known, ''we'' and others published many references to the relation of therapeutic and prophylactic vaccination. This term now has a much broader use, not only in the numeric sense as applying to various diseases, but in the sense of applying to a much larger variety of preparations, with very little detailed analogy. It is well known that a sharp practical distinction must be made between prophylaxis and therapeutics but the general principles underlying this distinction, the exact differences applying to various diseases from which germ products are theoretically or practically obtainable, and the discriminations necessary according to the exact kind of product employed, have not been satisfactorily worked out. Superficially considered, anything that will prevent, ought to relieve a disease, but this assumption is conspicuously and dangerously incorrect in the present instance. We suggest this subject to bacteriologists and chemists for discussion).

---

**Mesenteric Cyst With Torsion of Intestine.** Villar (ibid.) reports a case in which abdominal crises had occurred from an early age (present age not stated), with considerable intermissions, death occurring in the last crisis without intervention. A round, tough, mobile tumor of the size of an ostrich egg was found involving the middle of the transverse colon, attached by a pedicle of mesentery twisted on itself. Microscopic examination classified the cyst as serous. Loumeau considered it congenital, due to ectopy of the intestinal epithelial cells. Freche expressed the opinion that such cysts are analogous to the aberrant sebaceous glands sometimes found on the buccal mucosa.

